# Disruptions in Cancer Care Due to the COVID-19 Pandemic and Fear of Cancer Recurrence in Women with Breast Cancer: A Mixed-Methods Study

**DOI:** 10.3390/curroncol31020059

**Published:** 2024-02-01

**Authors:** Claudia Mc Brearty, Laurie Bisaillon, Michel Dorval, Hermann Nabi, Christine Desbiens, Julie Lemieux, Valérie Théberge, Amel Baghdadli, Sophie Lauzier, Josée Savard

**Affiliations:** 1 School of Psychology, Université Laval, Quebec, QC G1V 0A6, Canada; claudia.mc-brearty.1@ulaval.ca (C.M.B.); laurie.bisaillon.1@ulaval.ca (L.B.); josee.savard@psy.ulaval.ca (J.S.); 2 Université Laval Cancer Research Center, Quebec, QC G1R 3S3, Canada; michel.dorval@crchudequebec.ulaval.ca (M.D.); hermann.nabi@crchudequebec.ulaval.ca (H.N.); 3 Centre Hospitalier Universitaire (CHU) de Québec—Université Laval Research Center, Quebec, QC G1S 4L8, Canada; christine.desbiens@chudequebec.ca (C.D.); julie.lemieux@crchudequebec.ulaval.ca (J.L.); valerie.theberge.med@ssss.gouv.qc.ca (V.T.); amel.baghdadli@crchudequebec.ulaval.ca (A.B.); 4 Faculty of Pharmacy, Université Laval, Quebec, QC G1V 0A6, Canada; 5 CISSS de Chaudière—Appalaches Research Center, Lévis, QC G6V 3Z1, Canada; 6 Faculty of Medicine, Université Laval, Quebec, QC G1V 0A6, Canada; 7 Centre des Maladies du Sein, Centre Hospitalier Universitaire (CHU) de Québec—Université Laval, Quebec, QC G1S 4L8, Canada

**Keywords:** fear of cancer recurrence, breast cancer, COVID-19, pandemic, cancer care trajectory

## Abstract

Objective. This study investigated if fear of cancer recurrence (FCR) levels and the proportion of women having a clinical level of FCR differed by whether women had or had not experienced disruptions in their cancer tests and treatments due to the pandemic. Methods. We conducted a mixed-methods study between November 2020 and March 2021 among women diagnosed with breast cancer in the previous five years at the time of their entry in the study. Women completed a questionnaire online assessing disruptions in breast cancer tests and treatments due to the pandemic and the severity subscale of the Fear of Cancer Recurrence Inventory. Semi-structured interviews were also conducted with a subsample of 24 participants and were thematically analyzed. Results. The proportion of patients with a clinical level of FCR was significantly higher among those who experienced the postponement or cancellation of diagnostic and disease progression tests (e.g., blood tests, X-rays, or magnetic resonance imaging; adjusted PR = 1.27 95% CI = 1.13–1.43). Qualitative findings suggest that FCR was exacerbated by the pandemic context. In particular, perceived or actual barriers to care access due to the pandemic were identified as significant FCR-enhancing factors. Conclusions. These results highlight the need to keep diagnostic and progression tests as timely as possible to prevent increases in FCR levels and offer counselling about FCR when postponing or cancellation are inevitable.

## 1. Introduction

There is evidence that the coronavirus disease 2019 (COVID-19) pandemic has resulted in poorer mental health and increased levels of psychological distress in the general population [1]. One group that appears to have been particularly affected by the pandemic is cancer patients. Indeed, it has been shown that cancer patients experienced higher levels of anxiety and depression as compared to the general population and to pre-pandemic levels [2]. 

Early in the pandemic, expert groups issued recommendations concerning cancer management to optimize the fight against the pandemic and protect cancer patients who were considered to be at higher risk of complications from COVID-19 [3,4]. Canada applied international recommendations when indicated, to prioritize telemedicine consultations, postpone specific follow-up and imaging appointments, favor options minimizing hospital visits (e.g., oral rather than intravenous medication), and explore potential adjustments to treatment protocols to defer or spread out treatments (e.g., hypofractionation for radiotherapy). 

During the pandemic, a decrease in diagnostic mammograms [5,6] and cancer surgeries [7] has been documented. A study conducted in the United States among cancer survivors (n = 147) indicated that, in May 2020, 45.9% of the participants reported at least one provider-cancelled appointment [8]. Similarly, Papautsy and Hamish (2020) found that 44% of breast cancer survivors reported delays or changes in their cancer care trajectory (e.g., breast cancer surgery, diagnostic imaging and laboratory testing, anti-cancer therapies, and follow-up appointments). There is accumulating evidence that all of these disruptions had psychological consequences [2]. A Canadian survey conducted in 2021 among 413 cancer patients who had at least one appointment cancelled, postponed, or rescheduled, found that these disruptions had a significant impact on the mental and emotional health of 69% of the participants [9]. Similarly, in the French study COVIMPACT (n = 576), cancer patients who had experienced changes in their cancer care trajectory were more likely to report post-traumatic stress disorder symptoms [10].

Among the various psychological challenges faced by cancer patients during the pandemic, fear of cancer recurrence (FCR), defined as the “fear, worry, or concern regarding the possibility that cancer will come back or progress” [11], emerged as a particularly relevant construct. This relevance is heightened due to the central role that intolerance of uncertainty is believed to play in the etiology of FCR [12,13,14]. Uncertainty is inherent to the cancer experience, encompassing concerns about treatment effects, prognosis, and the potential for cancer recurrence [15]. The continuous state of uncertainty introduced by the pandemic, notably for cancer patients dealing with restricted access to treatment and periodic follow-ups, may have exacerbated their pre-existing levels of uncertainty and, consequently, their FCR. The general literature on FCR indicates that it is one of the most common unmet psychological needs in cancer patients and is associated with functional impairments, psychological distress, and a decreased quality of life [16]. FCR can occur at any time during the care trajectory: at diagnosis and during and after active treatment [17]. Although FCR can constitute a normal response to cancer, a substantial proportion of patients (22–87%) develop more severe or clinical levels of FCR [11,16]. 

While the prevalence of FCR is now relatively well documented in several subgroups of patients, it remains unclear whether and how pandemic-related stressors have influenced FCR levels. Results from a recent meta-analysis showed that the prevalence of clinically significant FCR was 67.4% among patients with cancer during the COVID-19 pandemic [18]. A scoping review of nine studies conducted in breast cancer patients indicated that treatment disruptions, such as delays and interruptions of the treatment plan, were associated with high FCR levels [19]. To our knowledge, no mixed-methods study has yet specifically examined the relationship between a wide range of disruptions in appointments and treatments for breast cancer and FCR. This relationship needs to be further studied, given that cancer patients believe that treatment and follow-up appointments are crucial moments to decrease or detect cancer recurrences and to be reassured [20,21]. Moreover, to better understand how disruptions in cancer care affect FCR levels, it is essential to ask patients directly about their experiences and perceptions using a qualitative methodology. 

The goals of this mixed-methods study were to: (1) assess if the FCR level differed whether or not women with breast cancer experienced the postponement or cancellation of medical tests/follow-up visits (i.e., follow-up mammogram and diagnostic and disease progression tests) or cancer treatment (i.e., surgery, radiation therapy, and chemotherapy), and the occurrence of other cancer care disruptions (i.e., change in the type of chemotherapy and shift to teleconsultation); (2) evaluate if the proportion of patients reporting a clinical level of FCR differed whether or not women experienced any of these disruptions during their cancer care; (3) explore the moderating role of several variables (i.e., time elapsed since diagnosis, treatment status, anxiety and depression levels) in the relationship between disruptions in cancer care and FCR; and (4) describe according to the participants’ perspective how disruptions induced by the COVID-19 pandemic affected FCR. It was expected that women who experienced disruptions in their care would show higher levels of FCR.

## 2. Materials and Methods

### 2.1. Design

The present study was carried out as part of a larger mixed-methods study aiming to describe the effects of the COVID-19 pandemic in women living in the province of Quebec (Canada) and diagnosed with breast cancer in the 5 years before their study entry. This study was conducted between November 2020 and March 2021, during the second wave of the pandemic in Quebec [22]. It was approved by the research ethics committee of the Centre hospitalier universitaire (CHU) de Québec-Université Laval (#2021–5344).

### 2.2. Participants

The inclusion criteria were: (a) female sex; (b) ≥18 years old; (c) having received a breast cancer diagnosis (all stages) in the past five years; (d) being able to understand French; (e) having access to the Internet; and (f) being a resident in the province of Quebec. 

Participants were recruited through advertisements via different platforms (e.g., Quebec Breast Cancer Foundation communication channels [email to members, Facebook page], CHU de Québec-Université Laval, and breast cancer Facebook pages). Online advertisements contained a REDCap link to assess eligibility and obtain electronic consent. Participants then completed questionnaires through the REDCap interface (quantitative study) and were asked if they agreed to be contacted to participate in a semi-structured interview (qualitative study). All questionnaires were administered in French. Given the scarcity of available literature in 2020 regarding the disruptions in cancer care due to the pandemic, we judged that 260 participants would allow us to capture the variability in experiences and assess the association between changes in cancer care trajectories and different psychosocial measures.

A sub-sample of patients who completed the questionnaires and agreed to be contacted again was invited to participate in an individual semi-structured interview (n = 24). They were selected to include women with different characteristics such as time since diagnosis, cancer stage (non-metastatic, metastatic) and living area (urban, rural). The interviews were performed by two interviewers (VM and LP) under the supervision of the principal investigator, who has expertise in qualitative research (SL). 

### 2.3. Measures

#### 2.3.1. Sociodemographic and Medical Characteristics

The research team developed a questionnaire to gather sociodemographic and medical information, including age, marital status, education level, cancer stage, and past and current treatments received. 

#### 2.3.2. Disruptions in Treatments and Cancer Care Appointments 

To assess disruptions in cancer care (independent variable), one item of the questionnaire probed participants about the direct impact the pandemic had (“We would like to know what impact the COVID-19 pandemic may have had on your breast cancer and your treatments. What events were directly caused by the pandemic?”). A list of pre-specified treatment and care disruptions was presented as follows, and respondents were asked to check anything that applied to their situation: (1) cancellation or postponement of a scheduled follow-up mammogram; (2) cancellation or postponement of scheduled follow-up appointments; (3) cancellation or postponement of a scheduled surgery; (4) cancellation or postponement of scheduled chemotherapy treatments; (5) change in the type of chemotherapy; and (6) cancellation or postponement of scheduled radiation therapy. In addition, two distinct questions assessed specifically whether patients had experienced cancellation or postponement of a diagnostic or progression test (e.g., blood tests, X-rays, or magnetic resonance imaging) or a shift to teleconsultation. 

#### 2.3.3. Fear of Cancer Recurrence Inventory

For this study, the nine items of the FCRI-severity (FCRI-S) subscale were used [23] (dependent variable). Items are rated using a 5-point Likert scale ranging from 0 (“not at all”) to 4 (“a great deal”), and 1 item is reverse-coded (item 13). A score of 13 or greater on the FCRI-S indicates a clinical level of FCR [24]. The original French-Canadian version was used and has demonstrated excellent psychometric properties (α = 0.95 ) [23]. 

#### 2.3.4. Personal Health Questionnaire Depression Scale (PHQ-8) 

This self-report questionnaire includes eight items and is used as a brief diagnostic and severity measure of depressive symptoms [25]. Each symptom is measured using a 4-point Likert scale ranging from 0 (“not at all”) to 3 (“nearly every day”). The total score ranges from 0 to 24. Scores of 5, 10, 15, and 20 indicate the thresholds for mild, moderate, moderately severe, and severe depression, respectively. In the context of chronic diseases, the reliability and validity of the PHQ-8 has been demonstrated, including a Cronbach’s alpha ranging from 0.82 to 0.85 [26,27]. To ensure consistency and comparability with prior research conducted in breast cancer patients during the pandemic, a cut-off score of 5 or higher was used to indicate a clinical level of depression [28,29]. 

#### 2.3.5. Generalized Anxiety Disorder 7-Item

This 7-item scale evaluates the frequency of the seven DSM-5 symptoms for a generalized anxiety disorder (GAD), over the past two weeks [30]. The 4-point Likert scale ranges from 0 (“not at all”) to 3 (“nearly every day”). Scores of 5, 10, and 15 indicate mild, moderate, and severe GAD symptoms, respectively. The reliability and validity of this scale have been established in cancer patients, with, for instance, Cronbach’s alpha ranging from 0.77 to 0.88 [31]. As in other studies conducted among breast cancer patients during the pandemic, a score of 5 or greater was used as a cut-off score to indicate a clinical level of anxiety [28,29].

#### 2.3.6. Semi-Structured Interviews

The guide for the semi-structured interviews focused on several dimensions of participants’ general experience during the COVID-19 pandemic (e.g., physical and mental health, social life, health-related behaviors) and the specific experience of having breast cancer during the pandemic. More specifically, we asked questions on how the pandemic affected breast cancer management (treatments, follow-ups, support from the healthcare team, and support from relatives), reactions regarding changes experienced, how the pandemic affected women’s perspective on breast cancer and potential remission, and factors that affected (positively or negatively) their experience. 

### 2.4. Analyses

#### 2.4.1. Statistical Analyses

Descriptive statistics (frequencies and means) were used to describe the participants’ sociodemographic and medical characteristics, the disruptions they experienced in their cancer care, as well as their level of FCR, anxiety, and depression. The associations of postponements, cancellations, or other modifications of tests or treatments with FCR was investigated using generalized linear models adjusted for age (treated as a continuous variable). For Objective 1, FCR was analyzed as a continuous variable using a normal distribution and an identity link. Mean FCR scores (and their 95% CI) were used to compare participants who had experienced or not a disruption in their care trajectory. An effect size was also calculated for each difference of means using the unadjusted pooled standard deviation (SD_pooled_) of the two groups being compared. To evaluate the association between disruptions in cancer care and clinical levels of FCR (Objective 2), prevalence ratios (PRs) and their 95% confidence intervals (Cis) were computed using a generalized linear model with a log link and a Poisson distribution. Model-robust variances were obtained with sandwich estimators to account for the larger variance of Poisson variables compared with binomial variables [32,33]. This model was chosen instead of a log-binomial model to avoid convergence problems. The same models were used for Objective 3 with the addition of an interaction term between the moderator (each moderator studied separately; i.e., time elapsed since diagnosis, treatment status, anxiety and depression levels) and the variable indicating if the cancer care trajectory was disrupted or not. Contrasts were used to compare the relationships between cancellations/postponements of tests and treatments or other changes (i.e., a change in the type of chemotherapy and shift to teleconsultation) and FCR between the categories of moderator. All available data were used in the analyses and all statistical analyses were performed with SAS 9.4, using a two-tailed alpha level of 5%.

#### 2.4.2. Qualitative Analysis

Interviews were audio-recorded and transcribed verbatim. The interviewers (LP—M.A. in sociology and VM—Ph.D. candidate in psychology) also produced individual summaries of each interview. These summaries were then discussed with the principal investigator (SL—M.A. in anthropology; Ph.D. in epidemiology), guiding the subsequent interviews and informing the analysis process. A thematic analysis of the integral transcripts was conducted using NVivo software (version 12; QSR International) [34,35,36]. The analyst (AB—M.Sc. in epidemiology) and a research assistant (AG—B.Sc. student in psychology) immersed themselves in the data by listening to the interviews, checking the transcript accuracy, and reading interviewers’ summaries. They developed a codebook using a mixed approach (inductive and deductive). In collaboration with the principal investigator, the analyst carried out data segmentation and categorization from the first four interviews and developed a preliminary codebook using the interview guide and allowing the emergence of codes from the corpus [37]. Then, the research assistant independently performed a validation exercise by proceeding to the data categorization from excerpts of the same interviews using the codebook. Their codifications were compared. In cases of disagreements, they reviewed and discussed the categorization until a consensus was reached. The principal investigator was consulted when an agreement could not be reached. The team (SL, AB, AG) refined the codebook. The analyst and research assistant independently tested this codebook on four other interviews and the remaining interviews were then coded. Relationships between the codes were explored and then grouped into potential themes. The analyst and research assistant summarized each potential theme, including relevant quotes. Three team members reviewed and named the potential themes, with the theme “cancer recurrence in the context of the pandemic” presented in this paper. To ensure the credibility and confirmability of the analysis, quantitative and qualitative findings from this study were triangulated [38]. The rigorous coding process and the examination of the findings by JS, an FCR expert not directly involved in the qualitative analysis, were used to establish dependability. Quotes were translated into English by a professional translator. 

## 3. Results

### 3.1. Demographic and Medical Characteristics 

A total of 245 participants completed the questionnaires. The participants’ characteristics are presented in Table 1. Patients were, on average, 52.9 years old (range: 26–77). Regarding the time elapsed since their breast cancer diagnosis, 31.1% had been diagnosed within the past year. A vast majority had undergone surgery (93.5%), while 71.4% and 62.9% had received radiation therapy and chemotherapy, respectively. At the time of data collection, most reported having no upcoming treatment (62.8%). 

### 3.2. Disruptions in Cancer Care 

Overall, 121 patients (51.5%) experienced at least one change in their cancer care trajectory due to the pandemic (see Table 2). Of these, 75 (31.9%) experienced the postponement or cancellation of at least one medical test or follow-up visit, such as a follow-up appointment, a follow-up mammogram, or a diagnostic and disease progression test (e.g., blood test, X-ray, or magnetic resonance imaging). Moreover, 17 patients (7.2%) reported at least one postponement or cancellation of treatment (i.e., surgery, radiation therapy, or chemotherapy) and 29 (12.3%) experienced the postponement or cancellation of both a treatment and a medical test or follow-up visit. 

### 3.3. FCR

On average, participants obtained a mean FCRI-S score of 17.9, which exceeds the clinical threshold (≥13; see Table 2). A large proportion of the patients (76.4%) reported a clinically significant level of FCR. 

### 3.4. Mean FCR Scores and Disruptions in Cancer Care

The results revealed that mean FCR scores were significantly higher among women who reported any postponement or cancellation of their medical tests, follow-up visits, or treatment (adjusted *M* = 18.9) as compared to those who did not experience any disruptions (adjusted *M* = 16.8), *p* = 0.03 (see Table 3). More specifically, participants who reported a delay or cancellation of a diagnostic and disease progression test had a significantly higher mean FCR level (adjusted *M* = 21.0) than those who did not (*M* = 17.0), *p* = 0.0002. However, no significant difference was found in mean FCRI-S scores whether women did or did not experience a postponement or cancellation of a follow-up mammogram, follow-up appointment, or surgery, or a shift to teleconsultation. The analysis could not be performed for postponement/cancellation of chemotherapy and/or radiation therapy and changes of chemotherapy type due to small numbers of these events. 

### 3.5. Clinical Levels of FCR and Disruptions in Cancer Care Trajectory

Overall, the results revealed that the proportion of patients showing a clinical level of FCR was not significantly different whether they did or did not experience the postponement or cancellation of at least one medical test/follow-up visit or treatment (adjusted PR = 1.11 95% CI (0.95–1.29; see Table 4). However, the proportion of women with a clinically significant level of FCR was 27% higher among patients who experienced a delay or cancellation of at least one diagnostic and cancer progression test than among those who did not (adjusted PR = 1.27 95% CI = 1.13–1.43). No other significant differences were found whether women did or did not experience any other type of change in their care trajectory (i.e., postponements or cancellations of follow-up mammogram, follow-up appointment, and surgery; and shift to teleconsultation) and again, due to the small numbers of events, the analyses could not be performed for postponement/cancellation of chemotherapy and/or radiation therapy and changes in type of chemotherapy.

### 3.6. Moderating Variables of the Relationship between Disruptions in Cancer Care and FCR

#### 3.6.1. Mean FCR Scores

Moderation analyses were conducted with the only independent variable for which FCR mean scores significantly differed, i.e., delay or cancellation of diagnostic and disease progression tests. A significant moderating effect was found where the effect of a postponed or cancelled diagnostic and disease progression test on FCR levels was greater in women receiving or about to receive a cancer treatment (hormone therapy excluded) (*MD* = 7.6, *p* = 0.0001) than in those who had completed their treatments (*MD* = 2.6, *p* = 0.05; interaction *p* = 0.04). No moderating effect was found for the following variables: time elapsed since diagnosis (one year or less vs. more than a year, *p* interaction = 0.08), clinical levels of depression obtained on the PHQ-8 (clinical vs. sub-clinical levels, interaction *p* = 0.40), and clinical levels of anxiety obtained on the GAD-7 (clinical vs. sub-clinical levels, interaction *p* = 0.61). 

#### 3.6.2. Clinical Levels of FCR

Again, moderation analyses were performed using delay or cancellation of diagnostic and disease progression tests, the only independent variable for which the proportion of patients with clinical levels of FCR differed. Anxiety was the only significant moderator. Specifically, the effect of postponed or cancelled diagnostic and disease progression tests on the proportion of patients with a clinical FCR level was significantly greater in women with a subclinical level of anxiety (49% higher when experiencing a postponement or cancellation, *p* = 0.001), than in those having a clinical score on the GAD-7 (*p* = 0.14; interaction *p* = 0.02). No moderation effect was found for the presence of a clinical level of depression (interaction *p* = 0.33) or the treatment status (completed or not; hormone therapy excluded) (interaction *p* = 0.70), and moderation analyses could not be performed for time since diagnosis because some cells were too small.

### 3.7. Qualitative Analyses

The characteristics of women who participated in the semi-structured interview are presented in Table 1. Most participants reported having persistent fears about cancer recurrence or death in general (quotes #1–3; see Table 5). For some, the pandemic was perceived as an additional stressor to their already stressful cancer situation, because of the FCR (quote #4). Overall, women did not explicitly mention that the delays and postponements of treatment amplified their FCR, except for one woman who shared that delays in follow-up visits were a cause for concern and contributed to her FCR (quote #5). Some women noted that the pandemic context led to fears related to the evolution of the disease because they perceived that access to hospital care was limited due to sanitary measures and offloading of medical activities (quote #6), that access to health professionals was difficult (quote #7), or because they avoided using health care services despite their symptoms in order not to overload them (quote #8). One participant noted that telephone appointments were less effective in providing reassurance with regards to her FCR (quote #9). Some women stated that every year without a recurrence matters and that the pandemic did not allow them to fully enjoy being healthy during that time (quotes #10–11), whereas one woman deplored not being able to turn to her social network to get distracted from her FCR (quote #12). For one woman, the fear of COVID-19 was more important than her FCR (quote #13). Finally, one woman observed that, during the pandemic, she was no longer the only one afraid of dying and experiencing uncertainty about her health, and now these feelings were shared by society as a whole, in the face of the pandemic threat (quote #14).

## 4. Discussion

This study, conducted in women diagnosed with breast cancer, assessed the relationship between disruptions in cancer care trajectory due to the COVID-19 pandemic and FCR. It was postulated that FCR mean levels and the proportion of women with clinically significant FCR would be significantly higher among women who had experienced disruptions than those who did not. This hypothesis was supported by the association found by Kállay et al. [19] between elevated FCR levels and treatment disruptions during the COVID-19 pandemic, along with insights from FCR etiological models [12,13,14,19]. The results of the current study partially corroborated this hypothesis and showed that both mean FCRI-S scores and the proportion of women with clinical FCR were significantly higher in women who had experienced a delay or cancellation of diagnostic and progression tests.

Overall, our study findings indicate that FCR is an important psychological issue for women living with breast cancer in a pandemic context. Indeed, the results showed that the mean severity of FCR (FCRI-S; *M* = 17.9) fell within the clinical range and that more than three-quarters of the patients (76.4%) displayed a clinical level of FCR. This prevalence is higher than that obtained in pre-pandemic studies conducted in various patient subgroups (breast, lung, pancreatic, and endometrial cancer), which ranged from 53.1% to 60.1% using the same tool and cut-off score [39] and it was also higher than in a recent study conducted among 36 women treated for non-metastatic breast cancer in Quebec during the first wave of the COVID-19 pandemic, with 53% of women having clinically significant FCR [40]. The association of FCR with intolerance of uncertainty [41,42] may explain, at least in part, why FCR was so prevalent during the pandemic. Intolerance of uncertainty is a central construct in recent theoretical models of FCR etiology [12,13,14]. The COVID-19 pandemic added a great deal of uncertainty to a situation, a breast cancer diagnosis, that is already characterized by a high level of ambiguity, thus contributing to exacerbating FCR. 

In order to better understand why women with breast cancer were particularly likely to experience high levels of FCR during the pandemic, the present study aimed to determine whether disruptions to their breast cancer care could have played a role. Consistent with the initial hypothesis, the results showed that the level of FCR and the proportion of women with clinically significant FCR were significantly higher in women who had experienced a delay or cancellation of diagnostic and progression tests. Diagnostic and disease progression tests, such as blood tests, X-rays, and magnetic resonance imaging, are key procedures to detect cancer recurrences. Moreover, although studies tend to show that FCR increases in the moments preceding diagnostic and progression tests, it decreases when negative results are communicated [43,44,45]. Thus, these are pivotal moments for getting reassurance [20]. Data from the present study suggest that when patients are deprived of this reassurance due to external circumstances, such as the COVID-19 pandemic, levels of FCR are likely to increase. 

However, our results did not provide support for the hypothesis that FCR would be higher in women who experienced postponement or cancellation of cancer treatment. These findings are surprising and diverge from those reported by Kállay et al. [19] in a recent scoping review, where higher levels of FCR were associated with treatment interruptions and delays among women with breast cancer. Treatment postponement, as self-reported by patients, was infrequent in our sample (7.2%) and it is possible that treatments were postponed within a reasonable timeframe for patients and, as such, had less effect on their FCR levels. One may also consider that a large proportion of participants had completed their treatment at the time of the questionnaires’ completion. 

The moderation analyses indicated that the relationship between delays and cancellations of diagnostic and progression tests and FCR varied as a function of whether patients had completed or not their cancer treatment (except hormone therapy) and had clinically significant anxiety on the GAD-7 or not. Specifically, the relationship of postponement/cancellation of diagnostic and progression tests with FCR scores was significantly lower in women who had completed their treatments. These results seem at odds with the widely held assumption that FCR occurs after treatment completion but are consistent with some prospective studies, which showed that FCR is particularly high in the pre-treatment phase and reduces after treatment [46,47]. Besides, as already mentioned, a significant proportion of our participants completed the questionnaires several years post-treatment (i.e., up to 5 years post-diagnosis). Having tests that are postponed when medical follow-ups are already more spaced over time because of the absence of recurrence since treatment completion may be less disturbing psychologically. With regards to the moderating role of anxiety, it appears counterintuitive that the strongest association between delays/postponements of diagnostic and progression tests and the presence of a clinical level of FCR was found in women with a lower (subclinical) anxiety level. However, this could be because women with a clinical level of anxiety also had a higher baseline level of FCR whether or not they had disruptions in their medical tests, thus decreasing the possibility that experiencing such disruptions could impact their FCR level. Indeed, anxiety is a strong correlate of FCR [48,49,50]. 

The qualitative analysis findings provide contextual insights and complement the results of the quantitative analyses. Participants’ quotes affirmed the significance of FCR among women with breast cancer in general and underscored that the pandemic’s context, which was inherently characterized by a great deal of uncertainty, may have intensified this sense of uncertainty by “adding an extra layer on top of all other [cancer-related] stress and anxiety.” This could account for the heightened levels of FCR reported in this context. Consistent with the quantitative results, very few women explicitly mentioned that their FCR was exacerbated by treatment delays. However, the qualitative data contribute to a deeper understanding of the factors that could explain why FCR was so elevated during the COVID pandemic. In particular, the qualitative data underscore the potential impact of missed opportunities to provide reassurance to patients regarding cancer progression or recurrence. Indeed, the women mentioned that delays in appointments and follow-up examinations, or the shift to teleconsultation without a physical check-up, deprived them of the reassurance they typically get from medical consultations and tests. 

Moreover, the healthcare system’s overload was widely mediatized during the pandemic, contributing to increasing their uncertainty about accessing necessary care and services. Some women expressed fears of being unable to access care in the event of recurrence or other health issues, while others mentioned that they had refrained from seeking care to avoid adding to the system’s burden or contracting COVID. Finally, a few comments highlighted the importance of the social network as a crucial resource for coping with stress. Social distancing measures strongly marked the pandemic in Quebec, and according to some women, the ensuing isolation also deprived them of the social support they needed to better cope with their worries, including FCR. 

A strength of this study resides in having seized the opportunity of the pandemic to document the experiences of women with all stages of breast cancer at various points of their cancer trajectory (up to 5 years since diagnosis). The use of an empirically validated measure of FCR and of a clinical cut-off score constitutes another strength of the present study. Although a lack of consensus remains in the literature regarding the best clinical threshold to use for the FCRI, it was determined using a rigorous methodology [23]. 

Nonetheless, some limitations need to be acknowledged. First, the response rate for this study cannot be estimated since women with breast cancer were recruited through advertisements via different platforms of patient organizations. Second, a selection bias cannot be ruled out as it is possible that women who agreed to participate in this study were either more or less affected psychologically by the pandemic. This may have influenced the prevalence and severity of FCR. Third, it cannot be ruled out that some patients opted to postpone specific tests and treatments on their own accord. Nevertheless, we believe most disruptions resulted from changes in cancer care enforced by hospitals. Indeed, the types of disruptions that we assessed align with the recommendations issued by expert groups concerning cancer management during the pandemic. Another limitation of this study is its cross-sectional nature, which limits the causal inferences that can be drawn. Hence, it is impossible to conclude with certainty whether FCR really increased because of stressors associated with the COVID-19 pandemic. Finally, the statistical power of some analyses was limited due to the low occurrence of certain types of disruptions in the care trajectory (e.g., postponement/cancellation of chemotherapy and/or radiation therapy specifically). 

## 5. Conclusions

The findings of this study suggest that cancer patients are more likely to report high FCR levels in the context of a pandemic. More specifically, the postponement or cancellation of diagnostic and disease progression tests, as experienced by 22.9% of the patients, was associated with a higher level of FCR, especially in women who had not completed their primary cancer treatment. Together, these results contribute to a better understanding of the psychological consequences of a pandemic in women treated for breast cancer and highlight the need to keep diagnostic and progression tests as timely as possible to prevent increases in FCR levels. In a broader context, the findings of this study may also be helpful to guide research and clinical practice in the context of future crises in general, including other epidemics, but also environmental challenges [50]. Given that effective cancer prevention, diagnosis, and treatment involve multiple visits to healthcare facilities, individuals with cancer are particularly vulnerable to the impacts of a crisis on their access to essential care. Cancer centers should draw lessons from the pandemic and prepare themselves for the potential repercussions of future crises on healthcare delivery. One aspect of this preparation highlighted by our study is ensuring that healthcare professionals have the necessary skills to identify signs of FCR in patients, address their concerns, provide reassurance about their cancer care trajectory, and refer patients, when needed, to evidence-based interventions, such as group cognitive-behavioral therapy for FCR [15].

## Figures and Tables

**Table 1:** Participants’ demographic and medical characteristics.

	Entire Group (N = 245)		Participants in the Semi-Structured Interview (n = 24)	
Characteristics	*M (SD)*	n (*%*)	*M (SD)*	n (*%*)
Age (range: 26–77)	52.9 (11.4)		54.3 (12.6)	
Household type				
Living alone		50 (20.4)		6 (25.0)
Couple		170 (69.4)		17 (70.8)
Single-parent family		20 (8.2)		1 (4.2)
Other		5 (2.0)		0 (0)
Education				
College or less		132 (53.9)		11 (45.8)
University		113 (46.1)		13 (54.2)
Current occupation				
Full-time or part-time work		81 (33.9)		3 (12.5)
Sick leave		68 (28.5)		8 (33.3)
COVID-19 layoff		2 (0.8)		0 (0)
Retired/unemployed		60 (25.1)		9 (37.5)
Other		28 (11.7)		4 (16.7)
Missing		6		0
Annual family income (Canadian dollars)				
≤$59,999		49 (24.0)		6 (25.0)
$60,000–79,999		29 (14.2)		1 (4.2)
$80,000–99,999		24 (11.8)		8 (33.3)
≥$100,000		72 (35.3)		6 (25.0)
Did not know/did not want to answer		30 (14.7)		3 (12.5)
Missing		41		0
Time since the most recent cancer diagnosis (years)				
0–1 ^a^		76 (31.1)		10 (41.7)
1–2 ^a^		61 (25.0)		8 (33.3)
2–3 ^a^		48 (19.7)		2 (8.3)
3–4 ^a^		30 (12.3)		3 (12.5)
4–5		29 (11.9)		1 (4.2)
Missing		1		0
Cancer stage				
1–3		194 (79.2)		16 (66.7)
4		16 (6.5)		4 (16.7)
Did not know/unsure		35 (14.3)		4 (16.7)
Received cancer treatments ^b^				
Surgery		229 (93.5)		23 (95.8)
Radiation therapy		175 (71.4)		19 (79.2)
Chemotherapy		154 (62.9)		15 (62.5)
Hormone therapy		149 (60.8)		15 (62.5)
Targeted therapy		44 (18.0)		4 (16.7)
Ovarian function suppression		40 (16.3)		4 (16.7)
None		3 (1.2)		0
Current cancer treatments ^b^				
None		73 (30.2)		6 (26.1)
Hormone therapy		134 (55.4)		13 (56.5)
Other		69 (28.5)		9 (39.1)
Missing		3		1
Upcoming cancer treatments ^b^				
None		142 (62.8)		14 (63.7)
Hormone therapy		50 (22.1)		5 (22.7)
Other		76 (33.6)		8 (36.4)
Missing		19		2
Comorbid chronic diseases ^b^				
None		114 (53.0)		7 (36.8)
≥1		101 (47.0)		12 (63.2)
Missing		30		5
PHQ-8 total score (range: 0 to 24)	5.8 (4.8)		6.17 (5.0)	
None (0–4)		104 (47.3)		
Mild (5–9)		74 (33.6)		
Moderate (10–14)		31 (14.1)		
Moderate-severe (15–19)		6 (2.7)		
Severe (20–24)		5 (2.3)		
Missing		25		
GAD-7 total score (range: 0 to 21)	5.0 (4.7)		4.79 (4.9)	
Minimal (0–4)		119 (54.3)		
Mild (5–9)		67 (30.6)		
Moderate (10–14)		22 (10.0)		
Severe (15–21)		11 (5.0)		
Missing		26		

^a^ Excluding the upper bound. ^b^ The sum of these percentages exceeds 100% because some patients had received, were receiving, or were about to receive more than one treatment. Note. PHQ-8 = Patient Health Questionnaire—8 items; GAD-7 = Generalized Anxiety Disorder—7 items.

**Table 2:** Descriptive statistics on changes in the cancer care trajectory and FCR.

Characteristics	*M (SD)*	n (%)
**Postponement or cancellation (overall)**		
At least one medical test or follow-up visit postponed or cancelled		75 (31.9)
At least one treatment postponed or cancelled		17 (7.2)
Postponement or cancellation of both treatment(s) and medical test(s) or follow-up visit(s)		29 (12.3)
No postponement or cancellation of any type		114 (48.5)
Missing		10
**Postponement or cancellation of medical tests**		
Mammograms		
Postponed or cancelled		37 (15.7)
No postponement or cancellation		198 (84.3)
Missing		10
Follow-up appointments		
Postponed or cancelled		89 (37.9)
No postponement or cancellation		146 (62.1)
Missing		10
Diagnostic and progression test ^a^		
Postponed or cancelled		54 (22.9)
No postponement or cancellation		182 (77.1)
Missing		9
**Postponement or cancellation of cancer treatment**		
Surgery		
Postponed or cancelled		38 (16.2)
No postponement or cancellation		197 (83.8)
Missing		10
Radiotherapy and/or chemotherapy		
Postponed or cancelled		9 (3.8)
No postponement or cancellation		226 (96.2)
Missing		10
**Other changes in the cancer care trajectory**		
Changes in chemotherapy type		
Yes		3 (1.3)
No		232 (98.7)
Missing		10
Teleconsultation		
At least one remote consultation		206 (88.0)
None		28 (12.0)
Missing		11
**FCR symptom severity**		
FCRI-S total score (theoretical range: 0 to 36)	17.9 (7.5)	
Nonclinical levels of FCR (0–13)		55 (23.6)
Clinical levels of FCR (13–35)		178 (76.4)
Missing		12

^a^ Blood tests, X-rays, or magnetic resonance imaging. Note. FCRI-S = Severity subscale of the Fear of Cancer Recurrence Inventory.

**Table 3:** Mean FCR levels ^a^ and differences of means across cancer care trajectory changes.

Changes in the Cancer Care Trajectory	FCR Adjusted Mean ^a^	Mean Differences	95% CI	Effect Size ^b^	*p* ^d^
**Any postponement or cancellation**					
Yes	18.9	2.0	(0.2–3.9)	0.27	0.03
No	16.8				
**Postponement or cancellation of medical tests or follow-up visits**					
Postponement or cancellation of follow-up mammogram					
Yes	19.2	1.6	(−0.9–4.1)	0.21	0.21
No	17.6				
Postponement or cancellation of follow-up appointment					
Yes	18.8	1.5	(−0.4–3.5)	0.21	0.11
No	17.3				
Postponement or cancellation of diagnostic and disease progression tests					
Yes	21.0	4.0	(1.9–6.1)	0.55	0.0002
No	17.0				
**Postponement or cancellation of cancer treatment**					
Postponement or cancellation of surgery					
Yes	18.8	1.1	(−1.4–3.6)	0.15	0.39
No	17.7				
Postponement or cancellation of chemotherapy and/or radiotherapy ^c^					
Yes	18.7	-	-	-	-
No	17.9				
**Other changes in the cancer care trajectory**					
Change in chemotherapy type ^c^					
Yes	11.8	-	-	-	-
No	18.0				
Teleconsultation					
Yes	18.1	1.6	(−1.2–4.4)	0.21	0.26
No	16.5				

^a^ Age-adjusted. ^b^ Effect size = difference of means/SD_pooled_. ^c^ Cells were too small to perform the analyses. ^d^ *p*-value for Wald χ^2^ tests.

**Table 4:** Prevalence of clinical FCR levels ^a^ and prevalence ratios across cancer care trajectory changes.

Changes in the Cancer Care Trajectory	Prevalence of Clinical FCR (%) ^b^	Prevalence Ratio	95% CI	*p* ^d^
**Any postponement or cancellation**				
Yes	79.9	1.11	(0.95–1.29)	0.18
No	72.0			
**Postponement or cancellation of medical tests or follow-up visits**				
Postponement or cancellation of mammogram				
Yes	84.2	1.13	(0.96–1.33)	0.14
No	74.5			
Postponement or cancellation of follow-up appointment				
Yes	80.7	1.10	(0.96–1.27)	0.18
No	73.2			
**Postponement or cancellation of diagnostic and disease progression tests**				
Yes	91.1	1.27	(1.13–1.43)	0.0001
No	71.7			
Postponement or cancellation of cancer treatment				
Postponement or cancellation of surgery				
Yes	81.0	1.08	(0.91–1.28)	0.39
No	75.1			
Postponement or cancellation of chemotherapy and/or radiotherapy ^c^				
Yes	87.3	-	-	-
No	75.6			
**Other changes in the cancer care trajectory**				
Change in chemotherapy type ^c^				
Yes	31.2	-	-	-
No	76.7			
Telemedicine consultation				
Yes	76.6	1.02	(0.81–1.28)	0.88
No	75.3			

^a^ Age-adjusted. ^b^ Clinical cut-off score of 13. ^c^ Cells were too small to perform the analyses. ^d^ *p*-value for Wald *χ^2^* tests.

**Table 5:** Themes, sub-themes and quotes from participants.

Themes/Sub-Themes	#	Quote
1. FCR in general (not related to the pandemic)		
	1	“I tell you I’m always afraid it’ll come back (silence) the cancer […] Even if they tell me the cancer I had is extremely rare […] and that all the treatments that were needed to eliminate the cancer cells were done, there’s always the fear.” Patient #272
	2	“Even if they told me there was no chance of a recurrence…you always have that tiny little fly in the ointment, that you’re in a hurry to get the results.” Patient #388
	3	“Once the surgery is over and then your oncologist reassures you that the operation was a success, then the cancer is temporarily gone. I say temporarily because you’re in remission after surgery then you have a remission that lasts 5 years …sure in the back of your mind there’s always that fear of a cancer recurrence.” Patient #452
**2. FCR and the pandemic**		
2.1 Effect of the pandemic on FCR	4	“Interviewer: «So could you talk to me more in detail about the impact Covid had on your physical health?»Participant: (pause) There’s really pressure, also the stress that it will add an extra layer on top of all the other stress or anxiety inducing factors along with the fear of a recurrence which often comes in the first year (laughs) like all those things, it’s like there was an even bigger state of stress […] the thing is …it’s all these factors put together add an extra layer on top of the stress of having to check that everything is OK if I have my blood tests are OK, if I don’t have a recurrence somewhere, if a pain isn’t due to a bone metastasis, just another pain on top of all the others (laughs). The thing is it adds an extra layer to deal with […].” Patient #440
2.1.1 Disruption of care and FCR	5	“That’s it, the follow-ups were done, sure that worried me because when you have a follow-up at 6 months then it’s postponed, you know… that it’s a bit on my mind…as long, as long as you aren’t in full remission […] that really worried me because my follow-ups weren’t there were delays in the follow-ups and as long as you aren’t in complete remission from the cancer.. then well it’s worrying, I was afraid like “when they call me will they find something”. You know, it’s coz all my tests were postponed […] that was the thing, it was more having to deal with that […] that worried me because that’s the thing, if I hadn’t had cancer, I wouldn’t have been worried but […] as long as your 5 years aren’t up. It’s worrying when you know it’s been postponed.” Patient #110
2.1.2 Difficulties in accessing healthcare services and FCR	6	“Interviewer: “Ok, so at that moment what worried you particularly?” Participant: Well the results of the surgery has all the cancer gone away, the risk of a recurrence if there were after-effects of the surgery how were we going to proceed because the pandemic was gaining momentum […] they were closing some operating rooms. So it was really stressful because even if the surgery was successful we had to make sure the safety margin was all right […].” Patient #452
	7	«“Uhhm, it’s like worry, it was hard to talk to the specialists, I wasn’t able to talk to them.» But you know one fine morning, you’re not sick and then BANG […] Having to manage all that, sure the pandemic had a psychological impact, it was hard… it was hard. There was a fear of I was afraid that I would find myself with a problem I couldn’t solve by myself…that nobody could solve.” Patient #110
	8	“I won’t have it, I hope,… because I had…I have dizzy spells, I have a loss of vision. I have a cancer they say, that recurrences are often in the lungs, the brain, like…you know that’s it. It’s the constant worry and then I don’t want to bother them. I’m waiting for my appointment (laughter), I’m waiting (laughter). It forced me to tell myself that it is what it is, what will be will be and to leave it up to… like “whatever happens will happen”, if I have a brain tumour, if I have dizzy spells (nervous laugh)… like.. when they treat me and it’s too late, well that’s too bad then!” Patient #124
2.1.3 Changes in care delivery/interactions and FCR	9	“My fear of a recurrence, not of my boss, not of the pandemic. For me the fear is always present. I need to be reassured and of course on the telephone I find it’s not the same reassurance as in person (laughter)”. Patient #124
2.1.4 Perception of recurrence or death in the pandemic context	10	“Sorry for getting a little emotional (laughter) but that this year, which has been a year without a recurrence (emotional), well I…I would have liked to take advantage of it and I hope there will be many more but if there had been a recurrence it would have been, like, I’d lost one of those years when I could have had a good quality of life when instead I had to… […] I would say that when we’re more aware of our …our mortality that’s a year when you find it a little harder to have put everything aside.” Patient #440
	11	“Honestly I don’t know, I also try not to think about it too much…when we’re not out of the pandemic yet. I don’t even know if I’ll get throu... have the time to see a normal world again […] You know I can die in 3 years like I can die in 3 months, it can change fast. We didn’t talk about it but you know also the idea that I could die in a few days or in a few months not have a funeral.” Patient #23
2.1.5 Isolation due to the pandemic and FCR	12	“Like you always ask yourself if it will come back, what if there’s another can—that’s a question I asked my doctor, could it be that there’ll be another cancer, you know that’s the thing, that’s it-not being able to get your mind off it with other people.” Patient #189
**3. Fear of COVID-19 vs. FCR**		
	13	“Yes Covid scares me…it scares me a lot […] Almost more than my fear of a recurrence I believe.” Patient #124
	14	“Uhhm..like it was the unknown, I think it was also the same for everybody but I experienced it as if it were putting life on hold again but at the same time I think that [the pandemic] allowed me to be less afraid of a recurrence at the same time because (laughs) it made me laugh a little in the sense…I don’t know if this is normal (laughs)) no worries, but in fact I told myself we’re all in the same boat for the pandemic which came and nobody knew it was going to come so I told myself I wasn’t all alone anymore in being afraid (laughs) that something could happen in my life! If we’re all in the same boat now. Like it helped put everything in perspective and for me to be less worried about the possibility of a recurrence and I would say that that’s still a little the case because nobody knows uhhh..nobody really knows what will happen with all the variants and with cases that are starting to go up again, so I told myself like…wow, you know…like I’m not worse off than anybody else, we’re all in unknown territory right now […] I always told other people (laughs) like I told them “well now I’m not the only one who’s afraid to die, everybody’s afraid to die, it’s planetary, it’s global!” […] I don’t have to worry about the cancer anymore (laughing).” Patient #440

## Data Availability

The datasets generated during and/or analyzed during the current study are available from the corresponding author upon reasonable request.

## References

[1] Kumar A., Nayar K.R. (2021). COVID 19 and Its Mental Health Consequences.

[2] Chen G., Wu Q., Jiang H., Zhang H., Peng J., Hu J., Chen M., Zhong Y., Xie C. (2020). Fear of disease progression and psychological stress in cancer patients under the outbreak of COVID-19. Psycho-Oncology.

[3] Jazieh A.R., Chan S.L., Curigliano G., Dickson N., Eaton V., Garcia-Foncillas J., Gilmore T., Horn L., Kerr D.J., Lee J. (2020). Delivering Cancer Care During the COVID-19 Pandemic: Recommendations and Lessons Learned from ASCO Global Webinars. JCO Glob. Oncol..

[4] Al-Shamsi H.O., Alhazzani W., Alhuraiji A., Coomes E.A., Chemaly R.F., Almuhanna M., Wolff R.A., Ibrahim N.K., Chua M.L.K., Hotte S.J. (2020). A Practical Approach to the Management of Cancer Patients During the Novel Coronavirus Disease 2019 (COVID-19) Pandemic: An International Collaborative Group. Oncologist.

[5] Grimm L.J., Lee C., Rosenberg R.D., Burleson J., Simanowith M., Fruscello T., Pelzl C.E., Friedewald S.M., Moy L., Zuley M.L. (2022). Impact of the COVID-19 Pandemic on Breast Imaging: An Analysis of the National Mammography Database. J. Am. Coll. Radiol..

[6] Salem C., Hajj M.A., Kourié H., Haddad A., Khaddage A., Ayoub E.N., Jabbour K., Moubarak M., Atallah D. (2020). Radiology management of a ‘breast unit’ during COVID-19 pandemic: A single institution experience. Future Oncol..

[7] Canadian Institute for Health Information COVID-19’s Effect on Hospital Care Services. https://www.cihi.ca/en/covid-19-resources/impact-of-covid-19-on-canadas-health-care-systems/hospital-services.

[8] Finkelstein L.B., Fishbein J.N., Bright E.E., Nealis M., Schmiege S.J., Arch J.J. (2022). Cancer Survivors’ Cancellations of Healthcare Appointments During the COVID-19 Pandemic: Associations with Anxiety and Depression. Psycho-Oncology.

[9] Leger Impact of COVID-19 Crisis on Cancer Patients and Their Ability to Receive Treatment. https://survivornet.ca/wp-content/uploads/2021/08/Survey-3-CCSN-Impact-on-Cancer-Patients-3rd-Report_July-30-2021.pdf.

[10] Joly F., Rigal O., Guittet L., Lefèvre-Arbogast S., Grellard J.M., Binarelli G., Lange M., Rieux C., Fernette M., Tron L. (2021). Post-traumatic stress symptomatology and adjustment of medical oncology practice during the COVID-19 pandemic among adult patients with cancer in a day care hospital. Cancer.

[11] Lebel S., Ozakinci G., Humphris G., Mutsaers B., Thewes B., Prins J., Dinkel A., Butow P. (2016). From normal response to clinical problem: Definition and clinical features of fear of cancer recurrence. Support. Care Cancer.

[12] Lebel S., Maheu C., Tomei C., Bernstein L.J., Courbasson C., Ferguson S., Harris C., Jolicoeur L., Lefebvre M., Muraca L. (2018). Towards the validation of a new, blended theoretical model of fear of cancer recurrence. Psycho-Oncology.

[13] Savard J., Savard M.-H., Caplette-Gingras A., Casault L., Camateros C. (2018). Development and feasibility of a group cognitive-behavioral therapy for fear of cancer recurrence. Cogn. Behav. Pract..

[14] Savard J., Caplette-Gingras A., Casault L., Hains J. (2022). Treating Fear of Cancer Recurrence with Group Cognitive-Behavioral Therapy: A Step-by-Step Guide.

[15] Han P.K., Klein W.M., Arora N.K. (2011). Varieties of uncertainty in health care: A conceptual taxonomy. Med. Decis. Mak..

[16] Simard S., Thewes B., Humphris G., Dixon M., Hayden C., Mireskandari S., Ozakinci G. (2013). Fear of cancer recurrence in adult cancer survivors: A systematic review of quantitative studies. J. Cancer Surviv..

[17] Crist J.V., Grunfeld E.A. (2013). Factors reported to influence fear of recurrence in cancer patients: A systematic review. Psycho-Oncology.

[18] Zhang L., Liu X., Tong F., Zhou R., Peng W., Yang H., Liu F., Yang D., Huang X., Wen M. (2022). The prevalence of psychological disorders among cancer patients during the COVID-19 pandemic A meta-analysis. Psycho-Oncology.

[19] Kállay E., Medrea F., Dégi C. (2022). On top of that all, now COVID-19, too. A scoping review of specificities and correlates of fear of cancer recurrence in breast cancer patients during COVID-19. Breast.

[20] Brandenbarg D., Berendsen A.J., de Bock G.H. (2017). Patients’ expectations and preferences regarding cancer follow-up care. Maturitas.

[21] Malagón T., Yong J.H.E., Tope P., Miller W.H., Franco E.L. (2022). Predicted long-term impact of COVID-19 pandemic-related care delays on cancer mortality in Canada. Int. J. Cancer.

[22] Public Health Expertise and Reference Centre. Données COVID-19 par Vague Selon L’âge et le Sexe au Québec. https://www.inspq.qc.ca/covid-19/donnees/age-sexe.

[23] Simard S., Savard J. (2009). Fear of Cancer Recurrence Inventory: Development and initial validation of a multidimensional measure of fear of cancer recurrence. Support. Care Cancer.

[24] Simard S., Savard J. (2015). Screening and comorbidity of clinical levels of fear of cancer recurrence. J. Cancer Surviv..

[25] Kroenke K., Strine T.W., Spitzer R.L., Williams J.B., Berry J.T., Mokdad A.H. (2009). The PHQ-8 as a measure of current depression in the general population. J. Affect. Disord..

[26] Pressler S.J., Subramanian U., Perkins S.M., Gradus-Pizlo I., Kareken D., Kim J., Ding Y., Sauvé M.J., Sloan R. (2011). Measuring depressive symptoms in heart failure: Validity and reliability of the patient health questionnaire-8. Am. J. Crit. Care.

[27] Mattsson M., Sandqvist G., Hesselstrand R., Nordin A., Boström C. (2020). Validity and reliability of the Patient Health Questionnaire-8 in Swedish for individuals with systemic sclerosis. Rheumatol. Int..

[28] Cui Q., Cai Z., Li J., Liu Z., Sun S., Chen C., Wang G. (2020). The psychological pressures of breast cancer patients during the COVID-19 outbreak in China—A comparison with frontline female nurses. Front. Psychiatry.

[29] Juanjuan L., Santa-Maria C.A., Hongfang F., Lingcheng W., Pengcheng Z., Yuanbing X., Yuyan T., Zhongchun L., Bo D., Meng L. (2020). Patient-reported outcomes of patients with breast cancer during the COVID-19 outbreak in the epicenter of China: A cross-sectional survey study. Clin. Breast Cancer.

[30] Spitzer R.L., Kroenke K., Williams J.B., Löwe B. (2006). A brief measure for assessing generalized anxiety disorder: The GAD-7. Arch. Intern. Med..

[31] Esser P., Hartung T.J., Friedrich M., Johansen C., Wittchen H.U., Faller H., Koch U., Härter M., Keller M., Schulz H. (2018). The Generalized Anxiety Disorder Screener (GAD-7) and the anxiety module of the Hospital and Depression Scale (HADS-A) as screening tools for generalized anxiety disorder among cancer patients. Psychooncology.

[32] Lumley T., Kronmal R., Ma S. (2006). Relative Risk Regression in Medical Research: Models, Contrasts, Estimators, and Algorithms. UW Biostat. Work. Pap. Ser..

[33] Spiegelman D., Hertzmark E. (2005). Easy SAS calculations for risk or prevalence ratios and differences. Am. J. Epidemiol..

[34] Miles M.B., Huberman A.M. (2003). Analyse des Données Qualitatives.

[35] Patton M. (2015). Qualitative Research and Evaluation Methods: Integrating Theory and Practice.

[36] Braun V., Clarke V. (2006). Using thematic analysis in psychology. Qual. Res. Psychol..

[37] Paillé P. (1994). L’analyse par théorisation ancrée. Cah. De Rech. Sociol..

[38] Lincoln Y.S., Guba E.G. (1985). Naturalistic Inquiry.

[39] Smith A.B., Costa D., Galica J., Lebel S., Tauber N., van Helmondt S.J., Zachariae R. (2020). Spotlight on the Fear of Cancer Recurrence Inventory (FCRI). Psychol. Res. Behav. Manag..

[40] Massicotte V., Ivers H., Savard J. (2021). COVID-19 pandemic stressors and psychological symptoms in breast cancer patients. Curr. Oncol..

[41] Curran L., Sharpe L., MacCann C., Butow P. (2020). Testing a model of fear of cancer recurrence or progression: The central role of intrusions, death anxiety and threat appraisal. J. Behav. Med..

[42] Kyriacou J., Black A., Drummond N., Power J., Maheu C. (2017). Fear of cancer recurrence: A study of the experience of survivors of ovarian cancer. Can. Oncol. Nurs. J..

[43] Götze H., Taubenheim S., Dietz A., Lordick F., Mehnert-Theuerkauf A. (2019). Fear of cancer recurrence across the survivorship trajectory: Results from a survey of adult long-term cancer survivors. Psychooncology.

[44] Ozga M., Aghajanian C., Myers-Virtue S., McDonnell G., Jhanwar S., Hichenberg S., Sulimanoff I. (2015). A systematic review of ovarian cancer and fear of recurrence. Palliat. Support. Care.

[45] McGinty H.L., Small B.J., Laronga C., Jacobsen P.B. (2016). Predictors and patterns of fear of cancer recurrence in breast cancer survivors. Health Psychol..

[46] Sarkar S., Scherwath A., Schirmer L., Schulz-Kindermann F., Neumann K., Kruse M., Dinkel A., Kunze S., Balck F., Kröger N. (2014). Fear of recurrence and its impact on quality of life in patients with hematological cancers in the course of allogeneic hematopoietic SCT. Bone Marrow Transpl..

[47] Savard J., Ivers H. (2013). The evolution of fear of cancer recurrence during the cancer care trajectory and its relationship with cancer characteristics. J. Psychosom. Res..

[48] Zhang X., Sun D., Qin N., Liu M., Jiang N., Li X.L. (2022). Factors Correlated with Fear of Cancer Recurrence in Cancer Survivors: A Meta-analysis. Cancer Nurs..

[49] Montel S. (2010). Fear of recurrence: A case report of a woman breast cancer survivor with GAD treated successfully by CBT. Clin. Psychol. Psychother..

[50] Deimling G.T., Bowman K.F., Sterns S., Wagner L.J., Kahana B. (2006). Cancer-related health worries and psychological distress among older adult, long-term cancer survivors. Psychooncology.

